# Does the Self‐Myofascial Release Affect the Activity of Selected Lower Limb Muscles of Soccer Players?

**DOI:** 10.2478/hukin-2022-0050

**Published:** 2022-09-08

**Authors:** Tomasz Michalski, Tomasz Król, Piotr Michalik, Magdalena Rutkowska, Magdalena Dąbrowska-Galas, Damian Ziaja, Michał Kuszewski

**Affiliations:** 1 Department of Kinesitherapy and Special Methods, School of Health Sciences in Katowice, Medical University of Silesia in Katowice, Katowice, Poland.; 2 Department of Physiotherapy, School of Health Sciences in Katowice, Medical University of Silesia in Katowice, Katowice, Poland.; 3 Institute of Physiotherapy and Health Sciences, Jerzy Kukuczka Academy of Physical Education in Katowice, Katowice, Poland.

**Keywords:** electromyography, foam rolling, hamstring, soccer, myofascial self-release

## Abstract

Myofascial therapy has already become one of the basic forms of treatment of the locomotor system. One form of the therapy is Self-Myofascial Release, in which external force is applied to the body with the help of special rollers (foam rolling, FR). The aim of the study was to investigate the direct effect of Self-Myofascial Release of hamstring muscles using a foam roller on the bioelectric activity of selected muscles (biceps femoris and gluteus maximus) during squats. The study involved 40 male soccer players, who were randomly divided into two groups: experimental and control. The tests used did not show significant differences in the analyzed variables before the experiment (baseline measurement p > 0.05), while significant intergroup differences appeared for subsequent measurements, both for reference MVC values (p < 0.01 - for % gluteus maximus MVC, p < 0.001 - for % biceps femoris MVC) and for raw EMG values (p < 0.01 gluteus maximus and p < 0.001 - for % 0.0001 for biceps femoris). The use of self-myofascial release within the hamstring muscles leads to changes in the electrical potential of the muscles of the lower limb.

## Introduction

In the 21^st^ century, myofascial therapy has become one of the basic forms of treatment of the locomotor system. Working on dysfunction involving the myofascial tissue can have measurable effects on the functioning of the patient's locomotor system by optimizing the length of the muscle fibers and reducing the constant tension of the tendons (Schleip, 2003; Schleip et al., 2012). One form of the therapy includes self myofascial release (SMR), in which external force is applied to the body with the help of special rollers (foam rolling) characterized by different hardness, size and texture (Peacock et al., 2014). Research conducted by Curran et al. (2008) showed that the pressure force of the roller on the tissue and the surface area of contact of the roller with the body depend on the hardness of the roller and the texture of its surface. Studies conducted to date indicate that this type of myofascial relaxation can increase the range of motion (ROM) and reduce delayed onset muscle soreness (DOMS) after exercise (Halperin et al., 2014; MacDonald et al., 2013; Monteiro et al., 2018; Romero-Moraleda et al., 2017; Pearcey et al., 2015). The effects of using SMR can also depend on the time, intensity and speed of foam rolling, as well as the length of the interval between each rolling session (Monteiro et al., 2017a). There are many theories which attempt to explain the effects produced by the myofascial release, which also occur during SMR therapy. One of the basic theories underlying the mechanism of SMR is the structural change of fascial tissue after the supply of energy in the form of heat or mechanical deformation. As a result, there are changes in the adhesion of the tissue and the properties of the viscous and thixotropic fascia (Cheatham et al., 2015; Couture et al., 2015; Knight et al., 2001; Schleip, 2003). The influence of SMR on the thixotropic effect is short-lived, with tissues returning to their original state within few minutes of cessation of therapy, which explains the effect of immediate tissue release. To produce a longer thixotropic effect, a much longer or stronger stimulus is required than that provided when applying SMR (Schleip, 2003). The positive effects of SMR therapy can also be explained by changes in the neuromuscular system through stimulation of mechanoreceptors located in fascia (Schleip, 2003). Such an afferent stimulation affects the inhibition of muscle tone (Monteiro et al., 2018; Sullivan et al., 2013). Besides explanations focusing on the change in muscle tone, some authors postulate the effect of SMR on the change in muscle recruitment pattern, which does not affect changes in muscle strength (Ginszt et al., 2017; Halperin et al., 2014; MacDonald et al., 2013; Peacock et al., 2015). Arroyo-Morales et al. (2008) showed a decrease in the bioelectrical activity of the quadriceps muscle during a 40-min massage. Different duration in similar forms of therapy can decrease the Hoffmann's reflex. Available studies show that Hoffman's reflex is dependent on the depth of the therapeutic effect, the pressure exerted on the tissue during the massage (Goldberg et al., 1992; Morelli et al., 1999).

The main extensors of the hip joint are the hamstring muscles and the gluteus maximus (GM). The influence of the hamstring muscles on the knee joint depends on the current position of the hip joint. The tension of the hamstring muscles increases during flexion of the hip joint, while with the extension of this joint it decreases, which is also accompanied by a decrease in its effectiveness (Kapandji, 2011). Hamstring muscles generate a greater moment of force when they are in a partially elongated position, which is due to the component of particular muscles.

Disturbed work between hamstring muscles and the GM is one of the causes of pain in the lower spine. The delay in the activation of the GM in relation to the hamstring is the cause of pain in patients with lower back pain (LBP) (Hungerford et al., 2003); furthermore, a decrease in the strength of the GM occurs in patients with pain in the anterior part of the knee (Cichanowski et al., 2007; Hewett et al., 2006). The results of studies on the effect of SMR on muscle activity using a surface electromyographic signal (sEMG), are not completely clear. In a study comparing SMR to another form of stretching, researchers did not find significant differences in EMG activity between the techniques used (Worrell et al., 2001). Despite studies confirming the effect of SMR on mobility ranges and tissue properties, there are few studies confirming its effect on the value of the bioelectric signal during maximum isometric contractions (MVC) and sEMG activity. In studies using the roller for SMR, there were no changes in the moment of muscle force for the knee flexors and no changes in the antagonistic muscles, and only a moderate increase in the force of contraction of the knee flexors was observed (Gozubuyuk and Yucesoy, 2019; Macdonald et al., 2014). Also Sullivan et al. (2013) showed no effect of SMR applied to tendons, strength and speed indicators. There is also a confirmed negative effect of SMR on the strength of knee extensors. Researchers found that, depending on the number of foam rolling repetitions, the strength of the antagonists decreased (Monteiro et al., 2017b). There are also studies in which the use of a roller massager on the muscles of the sole of the foot, increased their MVC (Halperin et al., 2014). According to Mills et al. (2015), if during eccentric control of the torso flexion, which is a functional movement, the activity of the GM muscle decreases, then the co-activation of the hamstring muscles will increase. Such strenuous work of the hamstring can have a negative impact on its susceptibility to fatigue during physical activity and the ability to control the anterior tibial translatation of the knee in a weight-bearing position.

The aim of this study was to assess the impact of the intervention of SMR of the hamstrings on the activity of the biceps femoris and gluteus maximus muscles.

## Methods

### Participants

The study involved 40 soccer players of the regional soccer league. All the players were characterized by a high level of physical fitness and they regularly participated in soccer training, at least 3 times per week. They were randomly divided into 2 groups: experimental (A) and control (B). Group A included 20 men aged 25.5 ± 5.2 years. Of these, 17 players were field players and 3 goalkeepers. Group B included 20 men with the mean age of 26.3 ± 1.3 years, with the same number of players considering their playing position (17 field players and 3 goalkeepers). Basic characteristics of study participants are provided in Table 1.

**Table 1:** Basic characteristics of study participants

Variable	GROUP A				GROUP B			
	x̅	MIN	MAX	SD	x̅	MIN	MAX	SD
Age	25.5	20.0	30.0	5.2	26.3	20.0	25.0	1.3
Body height [cm]	177.9	168.0	190.0	5.7	177.8	169.0	188.0	4.3
Body mass [kg]	76.7	63.0	88.0	6.9	74.1	65.0	80.0	4.6
BMI	24.2	21.6	28.7	1.9	23.4	21.0	25.8	1.2

Both groups were homogeneous across all variables analysed. Exclusion criteria were the following: orthopaedic injuries in the lower extremities and the lumbosacral and lumbar complex within the last year, reported pain in the above mentioned body parts on the day of the study, myofascial therapy within the last 6 weeks and any non-specific neuromuscular disorders.

All participants were instructed to maintain their normal habits in terms of diet, physical activity and rest, and they were requested to abstain from excessive exercise for 48 h before the study.

All experimental procedures were approved by the Bioethical Commission of the Silesian Medical University in Katowice – decision number PCN/0022/kB1/147/i/19/20 of 03.03.2020.

### Test procedures

The sEMG evaluations were performed with the use of the MyoTrace 400 recorder (Noraxon USA Inc.) with a sampling rate of 1000 Hz. For sEMG testing, standard self – adhesive surface electrodes (Ag/AgCl) with a diameter of 10 mm were used, which were applied after preliminary skin preparation, i.e., removal of hair and washing with an alcohol solution. The activity of the muscles of the biceps femoris (BF) and the GM of the lower dominant limb was studied by the bipolar method, according to the standards of SENIAM (Surface Electromyography for the Non-Invasive Assessment of Muscle) (Merletti et al., 2001). For greater repeatability of the measurement, all electrode applications were performed by the same person who also recorded the measurements. The second investigator coordinated the MVC measurements and controlled the time without having insight into the results obtained by players. At the beginning, measurements of the MVC of the BF and GM were made. The participant performed three 4-s maximum isometric contractions separated by a 1 min rest interval.

The exercise during which the bioelectric activity of the BF and GM muscles was recorded, was a squat, performed by all subjects at the same pace (4 s per task: 2 s for the descending phase and 2 s for the ascending phase). A constant distance between the spaced feet and the knee joints for each of the players was assumed. In order to normalize the results obtained, each participant performed 3 attempts, and the average result of the maximum values obtained during each of them was a value related to the MVC value of the same muscle.

Applied Self-Myofascial Realease consisted of foam rolling on the hamstring muscles. The roller from Grid X (Trigger point, a division of Implus, LLC, 5321 IndustrialOaksBlvd., Austin, Texas, USA), high density, three-dimensional texture, covered with EVA foam, was used for this purpose. Participants were instructed to perform the procedure for 210 s. Players were instructed to move in the space between the sciatic tumor and the knee joint in both directions, bypassing the popliteal fossa, generating the greatest possible pressure of the limb on the roller under it. The rhythm of each repetition was 4 s (2 s in one direction and 2 s in the opposite one).

Immediately after foam rolling therapy, the sEMG of the muscles was recorded according to the same procedures as before the therapy. The time elapsed from the end of SMR application to the start of the exercise and at the same time to the sEMG measurements was the time required for re-application of the electrodes and averaged 62 ± 4.5 s. Five minutes after the cessation of the FR, the measurement was repeated.

**Figure 1:**
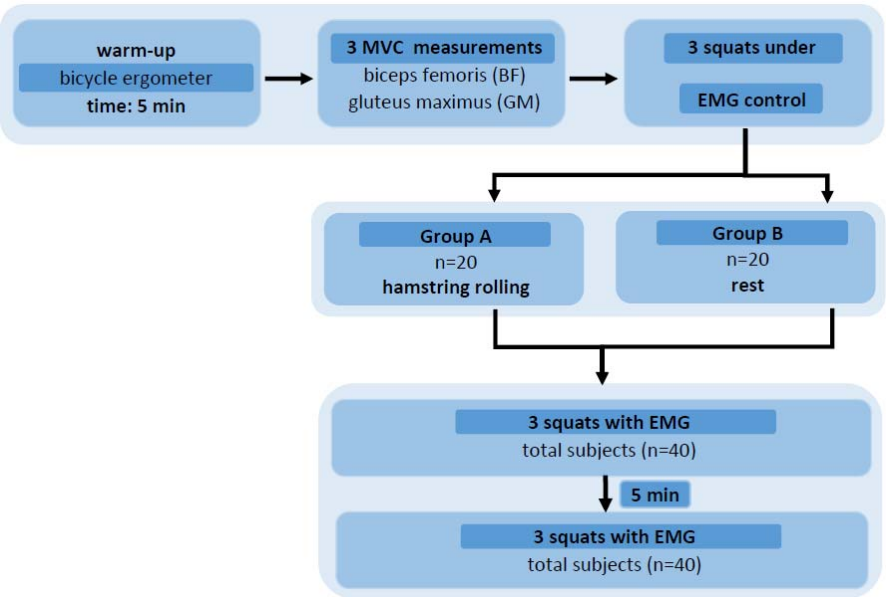
The experimental design

The experiment is presented in Figure 1.

### Statistical analysis

All results obtained in the study were collected in a database and then subjected to statistical analysis using Statistica 13 (TIBCO Softwear Inc.). A variance analysis test with repeated measurements was used and, when statistically significant differences were found, further analysis was carried out using the Tukey's post-hoc test for equal numbers.

## Results

The results of sEMG obtained during squatting were analyzed, as well as the results that were related to the values of MVC. These results were analysed as %MVC.

The tests used did not show significant differences in the considered variables before the experiment (baseline measurement, *p* > 0.05), while significant intergroup differences appeared for subsequent measurements, both for reference MVC values and for raw EMG values.

The post hoc analysis showed that for the results of %MVC, the largest differences for GM muscles were observed between the results in the group subjected to foam rolling immediately after the intervention (measure 2) and all the results of the control group (*p* < 0.05), in addition, a significant difference was observed between the values of the first measurement of the experimental group and the results of the second measurement of the control group (*p* < 0.05).

For the results of %MVC BF muscle, the largest differences were observed between the second measurement in the experimental group and all three results of the control group (*p* < 0.01), as well as in the values observed in the third measurement in the experimental group and all three measurements of the control group (*p* < 0.05). In addition, for the BF muscle, a significant difference also appeared between the values of the first measurement of the experimental group and the results of the second measurement of the control group (*p* < 0.05).

In both cases, no significant differences were observed between subsequent measurements in the experimental group.

For the raw EMG results for the GM muscle, the difference in the post-hoc test appeared only between the two groups in the result recorded in the second measurement (*p* < 0.05), while for the BF muscle statistically significant differences appeared in all measurements between the control group and the experimental group (*p* < 0.01).

Analysis of the differences in the studied variables, taking into account the position of the player on the field, did not show statistical significance.

Further details on the results are provided in Table 2.

**Table 2:** ANOVA results

Variable	BEFORE ROLLING		IMMEDIATELY AFTER		FOLLOW UP	
	A	B	A	B	A	B
%MVC GM	22.9	38.9	21.1++	41.2++	22.7	36.1
%MVC BF	21.7	27	20.7++	41.8++	23+	40.2+
RAW EMG GM	109.8	143	89.7++	153.7++	100	131.3
RAW EMG BF	114.8	113	109.9+++	237.7+++	121.3++	228.4++

Intergroup significant level: + *p* < 0.01; ++ *p* < 0.001; +++ *p* < 0.0001

## Discussion

The aim of this paper was to investigate the direct effect of SMR using a foam roller on the bioelectric activity of selected muscles during squats. The bioelectrical activity of the BF and GM was measured during the squat before and immediately after the intervention, as well as after 5 min of the intervention. The main finding of the study is that changes occurred both within the structures subjected to SMR, i.e., in the BF, and also in the GM where the SMR was not applied. It is worth noting that the durability of the effect obtained in both the measurements in the BF was much longer than the effect observed in the GM which disappeared within 5 minutes after SMR application. The results of this study are different from those of Beier et al. (2019) who studied the activity of the rectus femoris (RF) and GM muscles using sEMG during squats (10 repetitions with 70% of the maximum load for each subject). Their results obtained in comparison with the control conditions, which did not include the SMR, showed no changes in the bioelectrical activity of the muscles in the group that preceded the execution of squats with a two-minute automatic massage on the RF and GM. They found no significant differences in both mean peak and peak observed muscle activity, expressed as %MVC. Interestingly, they also showed no significant differences in the ROM of the knee joints of the subjects nor in their subjective assessment of the level of fatigue compared to the control condition (Beier et al., 2019). The observed differences with the results of this study may be due to the specificity of the study group, which consisted of eleven highly trained strength athletes, experienced in performing squats. The authors pointed out that training and the absence of myofascial restrictions could have caused the SMR to play less of a role in the effects on the ROM and muscle activation.

In another study with a similar profile, Bradbury-Squires et al. (2015) examined the activity of the vastus lateralis (VL) and BF during the lunge, before and after the roller-massager application (RM) on the quadriceps femoris muscle. They noted a 3% and 7% decrease in the VL muscle EMG during the exercise after five 20-s RM sessions and five 60-s RM sessions, respectively, on average for the entire exercise. The largest decrease in lateral extensive muscle EMG of about 24% was observed during the push-back phase. Those researchers did not notice changes in the BF muscle activity, and the decrease in VL muscle EMG was explained by an increase in neuromuscular performance during exercise. The results of that study differ from those presented in this paper, where no changes in muscle activity were observed in the experimental group before and after the intervention. Differences were found only when results were compared with the control group, which was not present in Bradbury et al.’s (2015) research. Differences in the test methodology, a different type of equipment used and the way it was used may have influenced the results of the tests. Bradbury et al. (2015) used a massage roller which was pressed against the participant's thigh with the device, always with the same force, corresponding to 25% of the body weight. In our study, a foam roller resting on a hard surface was used, which was pressurised by the weight of the participant. Different ways of applying self myofascial release can result in different forces exerted on the tissues by the automatic massage tool. Research by Baumgart et al. (2019) with 20 participants, showed that the average force with which the body pressed on the roller during the use of FR corresponded to 34 and 31% of the body weight for the front of the thigh and calf, respectively. At times, Baumgart et al. (2019) noted a pressure of more than 50% of the body weight. These values are higher than the constant pressure force that Bradbury et al. (2015) adopted in their study.

Several studies show that the difference between the results in the experimental group and the control group may be due to the effect of FR on the muscle spindles, as well as the Golgi tendon organs, which may contribute to changes in nerve activation (Behm et al., 2016). Stimulation of these mechanoreceptors would lead to a change in the activity of type IB nerves, which in a secondary way leads to inhibition of the activity of muscle spindles, thus leading to a greater feedback from the given muscle to the central nervous system (Jelerčič, 2019). Other authors (Macgregor et al., 2018) obtained a reduced EMG root mean squared (RMS) response during sub-maximal activity after FR. According to the authors, this reduced RMS protected the muscles from the effects of FR action. Similar results were obtained using massage techniques and static stretching where there was a decrease in spinal reflex excitability due to a probable change in excitability and Alpha motor neurons (Bradbury-Squires et al., 2015).

The limitations of the present study should be taken into account, which should certainly include the assessment of the effect of self-relaxation of the hamstrings on bioelectric activity during squats of only two muscles, i.e., the BF and GM. The significance of the study would increase if more muscle activity was observed in the lower extremities, since, as suggested by other authors, the therapy applied to the muscle may even affect the activity of the antagonists (Monteiro et al., 2017b). A single-limb therapy can also cause differences in muscle activity between both sides of the body (Ginszt et al., 2017). For this purpose, tests should be carried out on a more than two-channel EMG apparatus. Another limiting factor is the study group of only young men who were physically active at least 3 times per week. This specificity of the study group does not reflect variability in the general population. Further studies should also be carried out on women.

The observed change in the electrical potentials of the muscles after SMR can alter the excitability of the nervous system, which seems to be beneficial in the post-exercise recovery process. It seems reasonable to use rolling in the process of recovery, after intense exercise, but considering changes in the electrical activity of the muscles, caution should be used during the process of self-myofacial release before physical activity, especially before intensive exercise or competition. Interesting and surprising seems to be the fact that the effect of SMR appears temporary even in muscles not subjected to direct treatment. This can be extremely important in the process of recovery after very intense exercise where, due to very large post-exertion changes, direct therapy cannot be carried out. It turns out that the initial effect can be obtained by working on adjacent muscles located within the same myofascial anatomy chain. It is worth continuing research in this area, since it is a tool often used in sports and seems to have a beneficial effect on the athlete's body, however, our research does not answer how long the effect persists in the muscles subjected to the direct treatment.

## Conclusions

The use of self-myofascial release within the hamstring muscles leads to changes in the electrical potential of the muscles of the lower limb. These changes occur both within structures subjected to self-myofascial release (BF), and also in the GM where the SMR was not applied. The durability of the obtained effect is higher at the site subjected to the intervention.
